# From chat to act: large language model agents and agentic AI as the next frontier of AI in rheumatology

**DOI:** 10.1016/j.ero.2025.06.012

**Published:** 2025-07-29

**Authors:** Alfredo Madrid-García, Diego Benavent, Beatriz Merino-Barbancho

**Affiliations:** 1 Independent Researcher, Spain; 2 Rheumatology Department, Hospital Universitari de Bellvitge, Barcelona, Spain; 3 Escuela Técnica Superior de Ingenieros de Telecomunicación, Universidad Politécnica de Madrid, Avenida Complutense, Madrid, 30, Madrid, 28040, Spain

## Abstract

**Objectives:**

Large language models (LLMs) have begun to influence rheumatology, yet their static knowledge and hallucination risks limit their potential. Retrieval-augmented generation mitigates some limitations, but complex rheumatologic care demands real-time data access, multistep reasoning, and tool usage that exceed standard LLM capabilities. The objective of this study is to explore how agentic artificial intelligence (AI) can address the limitations of current LLM applications in rheumatology.

**Methods:**

We conducted a viewpoint analysis of the capabilities of agentic AI systems, focusing on their technical foundations, current use cases in healthcare, and relevance to the specific demands of rheumatologic care.

**Results:**

Agentic AI extends LLMs with planning, memory, and the ability to interact with external tools, enabling execution of complex tasks. These capabilities offer promising applications in rheumatology, including personalized treatment planning, automated literature synthesis, and clinical decision support.

**Conclusions:**

Agentic AI systems represent a necessary evolution to meet the complexity of rheumatologic care. Regulatory, ethical and technical challenges must be overcome before agentic systems can be safely deployed in routine rheumatologic care.

## INTRODUCTION

Transformer-based large language models (LLMs) are reshaping both research and clinical practice [1]. Their capacity for processing and generating human-like text has established them as major drivers for numerous applications including drug discovery and design, clinical decision support, patient communication and education or biomedical knowledge synthesis [2]. In specialities such as rheumatology, which are characterised by complex disease mechanisms, pronounced interpatient variability and large amounts of longitudinal, multimodal and often unstructured data from different sources [3], LLMs are becoming increasingly relevant [4]. Recent emerging applications of these models include educational question answering on medical licensing exams [5], clinical decision-support systems by integrating the European Alliance of Associations for Rheumatology (EULAR) and American College of Rheumatology (ACR) recommendation guidelines [6], rare rheumatic disease identification [7], crystal arthropathy detection, lupus classification [8,9] or providing patient education on antimalarial therapies [10].

Despite their proven capabilities, foundational LLMs have inherent limitations that pose challenges in medicine. Key limitations include a propensity for medical hallucinations [11], reliance on potentially outdated knowledge [12] and an inability to dynamically access real-time data or interact directly with external tools like databases or electronic health records (EHRs). Retrieval-augmented generation (RAG) architectures offer an efficient and reliable method for mitigating hallucinations and outdated knowledge [6,13,14]. However, the complexities of rheumatologic care and research often demand capabilities beyond the static input-output paradigm of standard LLMs, requiring dynamic interaction with auxiliary tools and data sources.

To overcome this limitation and enable more sophisticated applications in complex domains like rheumatology, artificial intelligence (AI) agents present a promising approach. Thanks to key technological milestones [15], these agents extend the capabilities of LLMs by equipping them with the ability to use external tools, plan and execute multistep tasks and interact dynamically with their environment [16]. In rheumatology, an AI agent could be designed not only to retrieve relevant research papers on, for instance, novel lupus nephritis treatments, but also to directly query a patient’s live EHR for the latest antidouble-stranded DNA titres, access clinical trial databases to find matching studies for a patient with refractory vasculitis based on specific inclusion/exclusion criteria, or even trigger alerts based on predefined clinical parameters to initiate subsequent actions (eg, a significant rise in C-reactive protein suggesting a follow-up appointment). Recognising this potential, we examine the specific opportunities presented by AI agents to improve care and research in rheumatic and musculoskeletal diseases (RMDs), alongside the associated challenges, exploring the key architectural components and potential applications in rheumatology. For clarity, a definition box summarising key terms is shown in Table 1 [4,6,17,18].

**Table 1:** Glossary of key concepts in agentic AI

Concept	Definition
LLM [4]	AI model trained on vast amounts of text data, enabling it to understand, process, and generate human-like language in response to prompts and questions
Hallucination [17]	A phenomenon where an LLM generates a response that is factually incorrect, yet presents it as if it were factual
Retrieval-augmented generation [6]	A technique that enhances the accuracy of LLMs by anchoring generative outputs to verified sources, thereby reducing the likelihood of hallucinations and improving clinical reliability
AI agent	An AI system that uses one LLM as its brain to autonomously reason, plan, and execute a sequence of actions to achieve a specific goal, by using external tools
Agentic AI	AI system that accomplishes a specific goal autonomously using more than one AI agent
Multi-agent system	A framework of multiple specialised AI agents that work together to solve a complex problem
Tool (AI agent context)	External resources that an AI agent can use to interact with the world, gather information and perform actions
Memory (AI agent context)	Short-term memory stores the current conversation; long-term memory is a persistent knowledge base that the agent can revisit across cases (eg, a patient’s longitudinal lab history)
Reasoning and planning	How an agent breaks a complex problem into steps, chooses actions and updates its plan as new information arrives
Constestable AI [18]	Contestable AI involves designing and building AI systems so that users and stakeholders can question, examine and steer their decision-making processes

AI, artificial intelligence; LLM, large language model.

### Agents and agentic AI

#### Introduction to agents

One of the companies that is leading the development of LLMs, OpenAI, defines agents as systems that independently accomplish tasks on your behalf [19]. In agentic AI, an agent or assistant is a computational system designed to *perceive* its environment (eg, free-text notes, laboratory results, medical-image data, research databases, drug leaflets; or tools accessible by application programming interfaces (APIs) or interoperability protocols such as model context protocol), *reason* over that information using an LLM, and *act* to accomplish a prespecified goal and *learn*.

Defining characteristics include proactivity and the ability to dynamically reason and plan the optimal sequence of actions to meet the objective. These agents can operate on a spectrum, from fully autonomous execution in well-defined tasks to systems incorporating human feedback or requiring explicit human approval for critical actions (ie, human in the loop), allowing for adjustable levels of control depending on the task’s nature and associated risks. For instance, an AI agent could, on receiving a new patient with suspected giant-cell arteritis, automatically retrieve the most recent erythrocyte-sedimentation-rate (ESR) measurement, screen the EHR for steroid contraindications and reserve an ultrasound slot, all without human intervention. AI agents exhibit varying degrees of autonomy in biomedical research. Some authors identified 3 levels distinguishing between LLMs functioning as tools, analysts and scientists [20]. Others have suggested 4 distinct levels based on their proficiency in hypothesis generation, experimental design and reasoning. These levels range from level 0, in which machine learning models serve merely as support tools for human scientists, to level 3, in which AI agents function akin to human scientists, capable of independently developing novel hypotheses and making pioneering discoveries [21]. In rheumatology, a level-0 agent might simply tag hand radiographs as erosive or nonerosive; a level-1 “DAS28 Assistant” could generate a focused, data-driven hypothesis, such as that ESR accounts for most of the variability in DAS28-ESR at follow-up, and perform basic regression analyses; a level-2 agent could mine multicentre longitudinal data across hospitals to propose that disrupted sleep patterns predict psoriatic arthritis flares, draft a multicentre trial protocol, simulate statistical power and iteratively refine the study design in collaboration with investigators; and a level-3 agent could integrate single-cell RNA-seq, spatial proteomics and imaging mass cytometry from early-rheumatoid arthritis (RA) synovial tissue to formulate de novo hypotheses that refine experimental approaches. However, these capabilities are not inherent to the agent itself. To perform a function such as identifying erosions on hand radiographs, the agent must rely on a clinically validated tool, such as a specialised machine learning model, that has followed a formal development lifecycle (ie, data collection, preprocessing, training, validation, deployment and monitoring).

Generative AI applications centre on producing novel content, not limited to text, based on their training data and input parameters [22], whereas agentic AI is optimised for decision-making, selecting, sequencing and executing actions toward a goal rather than for the creation of new artefacts [23]. While a foundational LLM provides the core engine for understanding and generating text, the agent architecture extends these capabilities by integrating components for task planning, external knowledge retrieval and tool invocation (eg, using APIs or databases). This addition transforms the foundational LLM into a system capable of structured, multistep decision-making and interacting effectively with its environment to achieve complex tasks in real-world applications [24].

#### Architecture of AI agents

**Figure 1:**
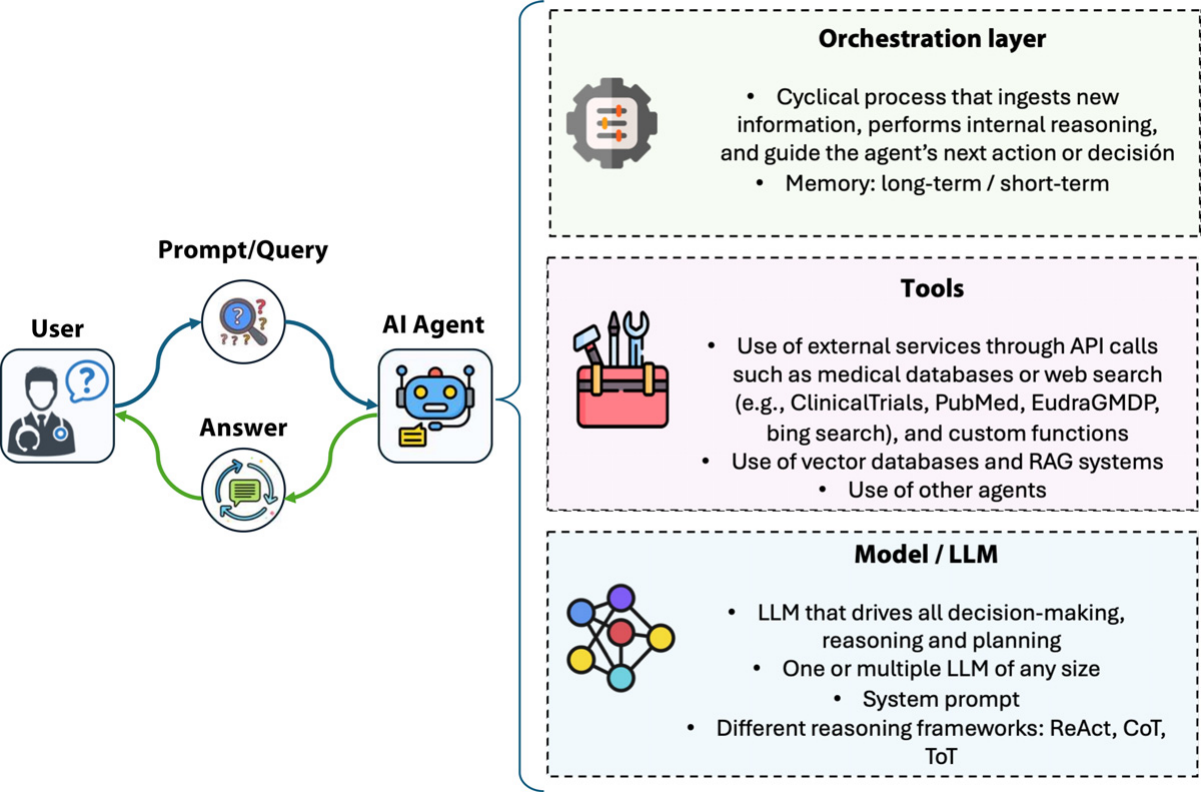
AI agent architecture, showing built-in capacity for planning and tool integration, which contrasts with traditional LLMs. AI, artificial intelligence; LLMs, large language models.

There is currently no single consensus on a standard architecture for AI agents. For clarity, this viewpoint adopts a common formulation that organises an AI agent into 3 principal components (Figure 1):

• Model: One or more LLMs serve as the decision engine. At each step, the model ‘routes’ the task, this is, it interprets the current context, decides what should happen next and, if needed, generates natural-language responses for the user.
• Tools: The model’s decisions are enacted by calling APIs, databases, clinically validated machine-learning models, code interpreters or other software utilities. These tools extend the agent’s reach into real-time EHR data, guidelines, clinical-trial registries, imaging pipelines or wearable-sensor streams.
• Orchestration layer: The orchestration layer manages the overall workflow and interaction between the model and the tools. This layer contains 2 main components:
  - Memory module: This module enables an agent to sustain coherent interactions and make informed decisions and actions based on past experiences and the current context.
  - Reasoning and planning module: This module determines the strategy to make decisions based on the available information.

This modular structure transforms a standard LLM into a dynamic system capable of multistep reasoning and interaction with its environment to achieve complex goals. A more detailed description of these architectural components and key reasoning strategies is provided in the Supplementary material *Architecture of AI agents* section, and in the Supplementary Tables S1 and S2.

#### Multiagent systems

Multiagent systems (MAS) introduce a further layer of complexity by employing multiple autonomous agents that interact within a shared environment. These agents can cooperate, coordinate, negotiate or even compete to solve problems or achieve goals that may be beyond the scope or efficiency of a single agent. In a MAS, agents often possess specialised roles or expertise and must communicate and negotiate to manage dependencies, resolve conflicts and synchronise their actions. This distributed approach allows for more robustness, scalability and the ability to model and tackle more intricate problems, such as coordinating diverse aspects of patient care pathways (eg, managing the journey of a patient with newly diagnosed RA from initial referral through to long-term biologic therapy monitoring) or simulating complex biological interactions relevant to rheumatologic diseases. Hence, in MAS, the focus shifts from a single decision maker (ie, agent) to a collective intelligence emerging from the interactions of multiple specialised entities (ie, agentic AI) [25]. For instance, recent work shows that aggregating the outputs of several LLMs can markedly boost diagnostic accuracy in clinical vignettes [26]. Different agents, each with a specific focus such as interpreting serologic assays like antineutrophil cytoplasmic antibody subtypes, analysing musculoskeletal ultrasound for synovitis or assessing patient-reported outcomes in spondyloarthritis, could engage in computational dialogue, mirroring the multidisciplinary team approach common in complex rheumatology cases. For example, the so-called MDAgents consist of a recently proposed framework that dynamically assembles a ‘care team’ of large-language-model agents, ranging from a solo primary-care-style agent to a full multidisciplinary panel, based on an upfront assessment of each query’s clinical complexity [27]. In rheumatology, MAS could coordinate renal-biopsy scheduling while a separate agent verifies drug-interaction alerts for advanced therapies.

**Figure 2:**
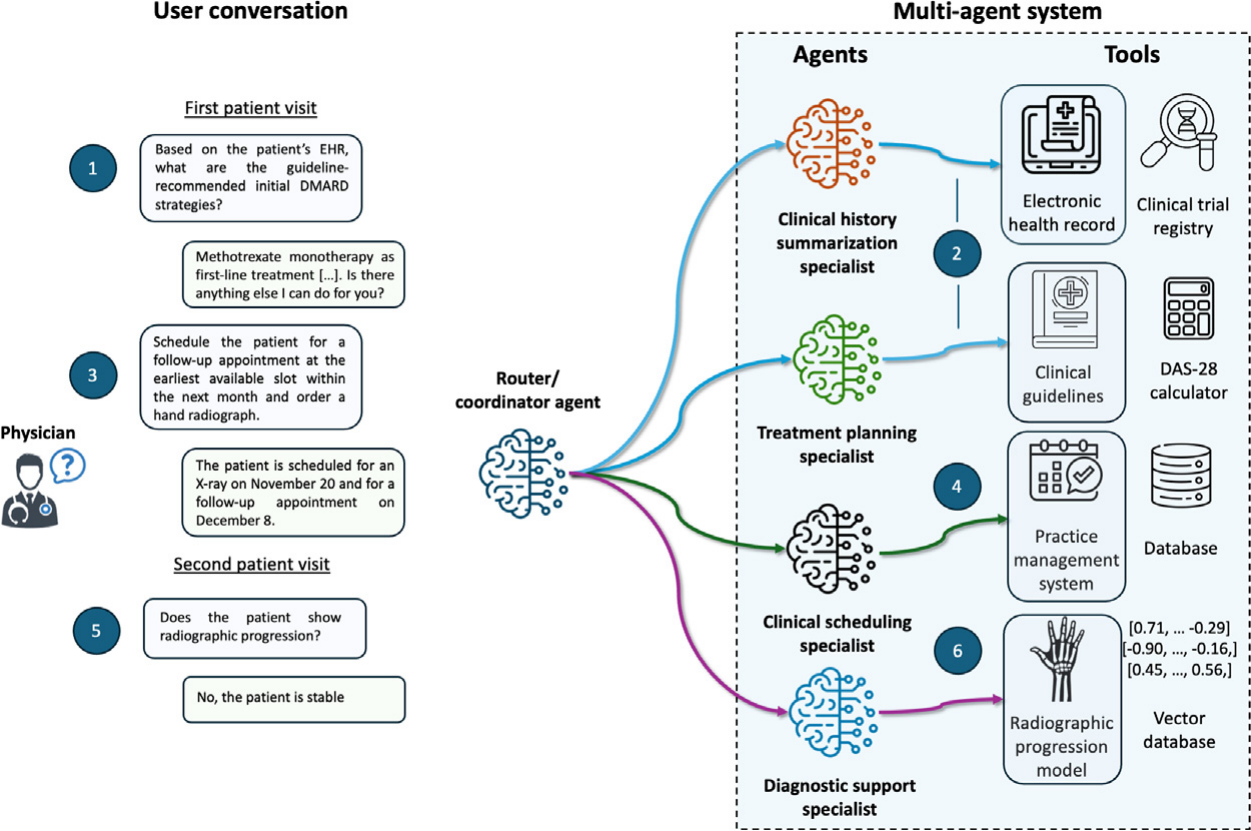
A hypothetical workflow of a multiagent system in a rheumatology clinical setting. This workflow demonstrates how a MAS can orchestrate multiple agents to perform complex, multistep reasoning, including the critical step of selecting the appropriate tool for each specific task. MAS, multiagent systems.

Figure 2 depicts a hypothetical MAS workflow in a rheumatology clinic. The system’s central coordinator agent, router, interprets the physician’s natural language prompts, decomposes them into executable subtasks and after reasoning and planning, delegates each to the most appropriate specialised agent. Every specialised, tool-equipped agent then reasons independently to select the optimal tool for its task. At the first visit (step 1), the physician requests guideline-recommended treatment strategies. The router activates the clinical history and treatment-planning agents, whose combined outputs yield the treatment recommendation (step 2). When the physician later asks to schedule a follow-up and a radiograph (step 3), the router hands the task to the clinical-scheduling agent, which books the appointment through the practice-management system (step 4). During the second visit, memory modules enable longitudinal management. Upon inquiry about radiographic progression (step 5), the router triggers the diagnostic-support agent, which runs the radiographic progression model on the new images (step 6) and concludes that the disease is stable.

This MAS workflow closely mirrors the collaborative dynamics of a human-multidisciplinary team. The system operates not as a single entity but as a coordinated group of specialists. In this analogy, the router acts as the lead clinician, triaging tasks to agents that emulate the roles of a consulting physician (treatment planning and clinical history specialists), a radiologist (diagnostic support specialist) and an administrative professional (clinical scheduling specialist). Just as in a clinical team, each agent contributes its specific expertise to achieve a common goal, from diagnosis to logistic management, illustrating how MAS can encapsulate and automate the complex and collaborative processes inherent in modern healthcare.

### AI agents in medicine

The application of agentic AI is emerging across various medical disciplines, demonstrating potential to address complex challenges in clinical practice and research, including in RMDs. A recent survey analysed 60 studies published between 2022 and 2024 on LLM-based medical agents, providing a comprehensive overview of their architectures, applications and challenges [24]. An analysis of key applications of AI agents in healthcare highlighted scenarios where they have been applied in different settings [28].

#### Clinical research methodology and drug discovery

TrialGenie is a 5-agent LLM framework that converts real-world EHR data into target-trial emulations and iterative protocol refinements, culminating in a comprehensive trial design report. By simulating a research team (ie, trialist, informatician, clinician, statistician and supervisor), TrialGenie tackles time-consuming tasks in a randomised controlled trial design like eligibility coding, cohort construction and causal analysis. Case studies using MIMIC-IV demonstrated strong performance on key tasks, suggesting potential to accelerate evidence-grounded trial design [29]. Concerning drug discovery, PharmaSwarm is a MAS designed to address high attrition rates and data fragmentation in therapeutic development. It comprises specialised LLM agents focused on different discovery modalities (eg, omics analysis, literature mining and market intelligence), integrated with tools for mechanistic simulation and interpretable binding prediction. It may facilitate a unified, hypothesis-driven workflow for proposing, validating and refining novel drug targets and compounds [30].

#### Genomics and translational medicine

TransAgent operates as a multiomics-aware AI agent using a ReAct-based architecture. It orchestrates over 30 epigenomic and transcriptomic tools. This system automates the transcriptional regulation analysis workflow, encompassing processes from raw sequencing data analysis (ChIP-seq and RNA-seq) to functional annotation and network modelling [31]. Besides, the clustered regularly interspaced short palindromic repeats (CRISPR)–generative pre-trained transformer (GPT) agent has been developed to automate and improve the design of complex CRISPR gene-editing experiments. It integrates curated domain knowledge with external computational tools (eg, for guide RNA and primer design) to determine next steps, and employs structured workflows based on state machines. This guides users through critical stages including CRISPR system selection, guide RNA design, delivery method recommendation, protocol drafting and validation strategy planning [32].

#### Patient communication

To address patients’ challenges in understanding complex medical reports accessible via EHR, an agentic LLM workflow using the reflexion framework was developed. This system translates radiology reports into patient-friendly language through iterative self-reflection, optimising for both medical accuracy and readability. Comparative testing demonstrated significantly improved accuracy and reduced need for manual revision compared with baseline LLM outputs, potentially enhancing the efficiency and reliability of patient communication [33].

#### Medical specialties

In radiology, RadCouncil framework has been introduced as a MAS-LLM designed to generate the impression section of chest x-ray reports from the findings section, addressing the challenges of manual reporting time, variability and hallucinations. This system employs 3 specialised, interacting agents—a retrieval agent using RAG to find similar reports, a radiologist agent to draft the impression using findings and retrieved examples and a reviewer agent to check consistency and provide feedback for iterative refinement [34]. Regarding oncology, GeneSilico Copilot has been proposed as an agentic platform that coordinates retrieval and reasoning tools. Functioning as a conversational clinical-decision copilot, it unifies breast-cancer guidelines, genomic profiles, drug information, clinical trial data and relevant biomedical literature to craft transparent and evidence-linked treatment recommendations [35]. In otolaryngology, ENTAgents has been introduced as an AI framework combining specialised agents coordinated through a task-planning approach that combines information from both internal knowledge bases, using RAG, and external sources like PubMed, ArXiv and web searches. Evaluations demonstrated that ENTAgents significantly improved accuracy, generated substantially more comprehensive and detailed responses compared to baseline LLMs and exhibited effective self-correction capabilities [36].

### AI agents in rheumatology

Although there are no published studies, to our knowledge, demonstrating the real-world implementation of fully autonomous AI-agentic systems in rheumatology, several independent strands of evidence show that the discipline has great potential for these systems to assist with complex data analysis, personalised treatment recommendations and patient monitoring, presenting numerous opportunities to improve patient outcomes and clinical workflows. Table 2 and Supplementary Table S3 summarise several potential applications of these agents in rheumatology.

**Table 2:** **Potential applications of agents in rheumatology**.

Application	Representative clinical scenario	Core AI capability^a^	Expected clinical impact
Diagnostic support	Automated grading of sacroiliitis on pelvic MRI in suspected axSpA	Vision-language model integrates images with clinical metadata to output score and diagnostic probability	Earlier and more reproducible axSpA diagnosis; reduced radiologist workload
	Classification of systemic sclerosis-specific antibody patterns from multiplex immunoassay	Pattern-recognition agent cross-validates serology with skin-thickness score	Precise subset assignment → tailored surveillance (eg, ILD risk)
Personalised treatment recommendation	Methotrexate initiation in RA incorporating particular genotype and baseline liver enzymes	LLM encodes ACR/EULAR algorithms plus pharmacogenomic rules	Optimised dose, fewer adverse events, faster time-to-remission
	Biologic choice in PsA with concurrent moderate-to-severe psoriasis and uveitis	Multiobjective optimiser weighs efficacy of different drugs across joints, skin and eye	One-shot selection of the most appropriate biologic, fewer treatment switches
Patient monitoring and self-management	Wearable-derived drop in gait speed and heart-rate variability predicting axSpA flare	Time-series transformer flags risk ≥7 days before clinical flare	Just-in-time tele-review; NSAID burst averts work absence
	Smartphone PASI capture guiding topical‐to-biologic escalation in psoriasis	On-device image classifier with federated learning	Objective, home-based disease-activity tracking; empowers patient
Clinical-trial optimisation	Screening for progressive fibrosing ILD in connective-tissue disease using HRCT reports + PFT trends	NLP + tabular fusion model applies inclusion/exclusion cascade	Triples eligible-patient yield while halving coordinator time
Care coordination and administrative automation	Multidisciplinary axSpA pathway (rheumatology, radiology and physiotherapy)	Agentic workflow engine synchronises appointments, shares structured notes	Shorter diagnostic delay and fewer missed physiotherapy sessions

ACR, American College of Rheumatology; axSpA, axial spondyloarthritis; HRCT, high-resolution computed tomography; ILD, interstitial lung disease; LLM, large language model; NLP, natural-language processing; PASI, Psoriasis Area and Severity Index; PFT, pulmonary-function test; RA, rheumatoid arthritis; SPARCC, Spondyloarthritis Research Consortium of Canada; NSAID, non-steroidal anti-inflammatory drugs; PsA, psoriatic arthritis; MRI, magnetic resonance imaging; EULAR, european alliance of associations for rheumatology.

a Examples of model families/toolsets: vision-language transformers, LLMs with retrieval-augmented generation, time-series transformers, NLP with rule-based reasoning engines.

Emerging studies demonstrate near-expert diagnostic accuracy for LLMs, prospective triage pipelines that reprioritise referrals and schedule investigations and continuous remote-monitoring loops that detect impending flares and draft medication adjustments [5,37,38]. A recent head-to-head study confirmed that GPT-4 outperformed 2 rival foundation models in subfields such as osteoarthritis and RA, highlighting the maturity of the underlying language engine [39]. Although these systems still function as ‘chatbots’, embedding them inside an orchestration layer that queries drug-interaction databases and guideline repositories converts a passive interface into an active treatment-planning agent capable of proposing and justifying personalised and guideline-concordant regimens.

Some relevant topics in rheumatology, such as drug-safety surveillance, fit naturally into an agentic workflow. Postmarketing pharmacovigilance for biologic/targeted synthetic disease-modifying antirheumatic drugs (DMARDs) relies on distributed data sources. An agent that continuously ingests spontaneous-report databases, longitudinal claims and local laboratory alerts can learn aberrant signal patterns faster than quarterly manual reviews, triage potential safety signals for clinician validation and automatically file validated events with regulatory authorities.

Besides, remote monitoring provides continuous context for agentic reasoning. Wearable sensors and smartphone assays show the ability to differentiate RA patients from healthy controls with high accuracy (F scores around 0.81) and stratify disease severity even more accurately when fused with patient-reported outcomes [40]. An agent equipped with this could potentially run a ‘treat-to-target’ loop: upon detecting a high-probability flare trajectory, it recommends a step up of treatment (under rheumatologist oversight) or an urgent appointment.

The convergence of these examples suggests a plausible near-term evolution in rheumatology: first, single-purpose ‘micro-agents’ embedded within existing EHR systems; next, cooperative multiagent constellations that mirror multidisciplinary teams; finally, fully equipped rheumatology operating systems that merge guideline reasoning and continuous monitoring into a closed-loop care platform.

### AI agents’ implementation in clinical care

The successful integration of agentic AI into routine rheumatologic care depends on both technical infrastructure and clinical workflows [41].

#### Technical infrastructure

A) Interoperability and access between AI agents and existing health-information systems are essential. Agents must securely access and interpret patient data in real time, requiring adherence to established data-exchange standards. Standards such as Fast Healthcare Interoperability Resources (FHIR), built on a representational state transfer (RESTful) application programming interface (API) architecture, let AI agents quickly retrieve structured patient data, accelerating their development and deployment in healthcare. Notably, FHIR adoption is growing in rheumatology [42]. Equally important is an agreement by health-information-system vendors to expose APIs, interfaces or services across disparate platforms as siloed agents are incapable of delivering clinically actionable insights. However, a significant barrier is the continued use of legacy systems that were not designed for modern data exchange and lack standardised APIs. Finally, emerging agent interoperability protocols are currently under development (eg, model context protocol) with the promise to standardise agent-to-system and agent-to-agent communication, enabling coordinated actions, dynamic data exchange and scalable integration across diverse clinical environments.
B) The flow of sensitive patient information must remain compliant with all applicable privacy regulations. Rigorous deidentification protocols, granular access controls and end-to-end encryption are required safeguards. General Data Protection Regulation (GDPR) and Health Insurance Portability and Accountability Act (HIPAA) frameworks establish legal and technical standards for data protection, mandating transparency, purpose limitation, data minimisation and patient consent, principles that must be embedded into the design and deployment of AI agents in healthcare. Recent framework proposals also leverage zero-knowledge proofs to enable compliance verification without exposing underlying patient data [43].
C) Scalable and reliable computational resources are needed [44]. Complex agentic workflows might demand either substantial on-premises hardware or cloud-computing environments that can accommodate intensive processing and the storage of large datasets. This is particularly challenging for low-resource settings with limited budgets for deployment and insufficient access to specialised IT personnel. Without dedicated funding for infrastructure upgrades and ongoing technical support, these institutions risk falling further behind, exacerbating inequities in access to advanced AI-driven care.

#### Clinical workflows

A) Governance and human oversight are indispensable. Clinical workflows must be designed to define the scope of agent autonomy, specifying which actions can be performed autonomously and which require human review and approval especially in high-stakes decisions, such as treatment plan modifications or medication ordering, in which the potential for error carries significant risk.
B) The introduction of AI agents also necessitates a re-evaluation of clinical roles, including structured upskilling for healthcare professionals. New protocols must guide how clinicians interpret and act on AI-generated recommendations, and comprehensive training should equip health professionals to recognise both the capabilities and the limitations of these systems, intervening whenever the agent’s output conflicts with clinical judgement.
C) Ensuring the transparency and explainability of AI agent actions is vital for building trust and facilitating safe clinical use. Clinicians must be able to understand the rationale behind an agent’s recommendations. The decision-making process should be interpretable, allowing for effective review and troubleshooting. Without this transparency, the adoption of agentic AI in routine clinical practice will face significant hurdles.

Once the foundational aspects are covered, the implementation of AI agents should begin with a manageable proof of concept (PoC) to measure the impact and effectiveness of the AI agent while minimising risks. The focus should be on low-risk applications in which the outcomes can be easily monitored and assessed. Human supervision is essential during this stage to guide the agent, validate its outputs and build trust in its capabilities. For instance, an initial PoC in a rheumatology clinic might focus on purely administrative or preparatory tasks such as history summarisation: the agent could process a patient’s entire medical record to generate a concise summary of their clinical history, previous treatments and relevant comorbidities for the clinician to review before a consultation or could triage incoming patient portal messages, selecting urgent queries for immediate human attention while autonomously handling routine requests like appointment confirmations or prescription refill notifications.

After successfully demonstrating the PoC’s viability, the AI agent’s scope could be expanded, integrating it more deeply with existing workflows. Examples include monitoring and alerting incoming laboratory results and patient-reported outcome measures, automatically flagging abnormal findings or clinically significant changes for rheumatologist review.

As the AI consistently proves its reliability and accuracy, the level of direct human supervision can be gradually reduced, paving the way for a full-scale rollout. High-risk applications, including diagnostic support or treatment planning, should only be considered after substantial experience has been gained and trust in the system has been firmly established.

## THE FUTURE OF AI AGENTS AND RHEUMATOLOGY

The anticipated growth and impact of agents are significant. Analysts project that by 2028, about one-third of all software applications will include agentic capabilities, whereas autonomous systems will handle roughly 15% of routine workplace decisions. In addition, 82% of companies expect to have integrated AI agents within the next 3 years [45]. Moreover, MAS has entered the Innovation Trigger phase of the 2024 Gartner Hype Cycle for AI, suggesting a potential timeframe of 5-10 years before widespread adoption [46]. Rheumatology is quietly amassing the data modalities, validated LLM capabilities and workflow integration points required for agentic AI. The absence of a headline ‘rheumatology agent’ in the literature should therefore not be read as a lack of progress but as evidence that the speciality is approaching an inflexion point in which today’s interoperable modules will be orchestrated into tomorrow’s autonomous collaborators. Although it is hard to forecast the specific extent and timeline for the adoption of AI agents for RMDs in the coming years, it is hypothesised that their integration into clinical practice will be heavily dependent on the development and implementation of clear regulatory frameworks in healthcare and the accumulation of robust evidence demonstrating their safety, efficacy and clinical utility. Despite this promising trajectory and its potential to enhance both clinical practice and research in rheumatology by automating complex tasks, significant concerns remain. These include unintended consequences or rogue actions arising from algorithmic bias or errors as well as ethical violations related to data privacy, algorithmic transparency and accountability in decision-making. Indeed, the ethical considerations inherent in the development and deployment of LLMs within AI are further magnified in the context of agents, primarily due to their fundamental attribute of autonomous decision making [47]. Accordingly, recent research on AI agents has focused on responsible AI [48], governance [49], ethics [50], fairness [51], risks [52] and challenges [28,53].

### Responsible AI agents

To be acceptable in clinical care and research, an AI agent must ensure that its actions consistently reflect both the immediate goals of the end-user (eg, a clinician, researcher or patient) and the broader ethical and societal values that govern healthcare practice. Achieving such value alignment requires agents to perform tasks in a helpful, honest and harmless manner, even in complex and dynamic environments. In this context, explainability and contestability become fundamental. The first ensures transparency by allowing users to understand the reasoning behind an AI agent’s decisions and actions (eg, allowing clinicians to review why a vasculitis alert fired before acting on it, ensuring that clinical decision-making remains transparent, accountable and aligned with best-practice care). The second, contestability, provides essential oversight, enabling users to question, dispute and potentially override the agent’s actions, such as allowing a clinician to flag and override an AI-suggested antibiotic escalation when it conflicts with the patient’s documented allergies. Both rely on robust explanatory capabilities. By clarifying the reasons behind past actions and outlining forthcoming steps in advance, they enable users to identify misalignments early, influence the agent’s behaviour and build a more reliable alignment with their preferences and operational expectations.

These mechanisms are essential to mitigate function-calling hallucinations, in which an agent incorrectly invokes a tool or misuses it, such as a disease activity score (DAS) calculator, in which the tender and swollen joint counts are inadvertently swapped.

Given that agents can act independently, establishing human accountability is essential. This requires responsible AI frameworks that assign responsibility to the organisations that design, deploy and profit from these agents. For instance, in a MAS setting, one agent may triage referrals, a second interprets musculoskeletal ultrasound and a third calculates weight-based methotrexate dosing. An error in the final step might harm a patient, yet each vendor points to another module in the chain. When no single party controls the full decision pathway, assigning legal and moral responsibility becomes contested.

### Governance and AI agents

Governance frameworks for rheumatology-focused AI agents must deal with information asymmetry (ie, an agent that drafts treatment plans could see more of the underlying data than the supervising clinician, placing the latter in a vulnerable position), ambiguous authority (ie, what APIs or medical information the agent has accessed to and how the agent can alter this information), conflicted loyalties (ie, AI agents developed by for-profit corporations may have objectives that are not aligned with patients’ welfare) and cascading delegation (ie, tool-using agents may call subagents such as imaging-analysis modules that are built by different vendors). Hence, conventional governance mechanisms may not be enough.

### Ethics

The ethical deployment of agentic AI in rheumatology necessitates careful consideration across several domains, including trust, misinformation, undue influence, fairness and bias, privacy, and intellectual property. Challenges within these domains are often linked to the agent’s foundational components: how the underlying LLMs were trained, the guardrails implemented, the degree of LLM alignment achieved, the characteristics of the training datasets used and the specific tools the agent is permitted to access. Bias inherent in any of these elements can inevitably lead to biased outputs from the agent [54]. Furthermore, the sensitive nature and longitudinal depth of rheumatologic data, often dispersed across various sources, necessitate robust privacy-preserving infrastructures. Additionally, because AI agents use multiple tools, the overall agentic system is ultimately only as strong as its weakest component, highlighting the need for careful integration and vetting of tools. Moreover, intellectual property considerations are crucial; preventive measures must be implemented within AI agents to avoid copyright infringement. As these models often have access to the internet and vast datasets, they might base their outputs on copyrighted material. Therefore, safeguards are required to ensure compliance with copyright law and to satisfy regulatory frameworks, such as the EU AI Act. This has led to new initiatives. A recent proposal calls for regulators to assess AI agents not merely by their training or inference compute, but by the extent of their autonomous action sequences, by limiting the length of unsupervised operations to the empirically validated safe threshold [55].

### Technical challenges

Despite the rapid progress of agentic AI, several technical hurdles must be addressed in the coming years. Among them are scalability, ensuring that systems can reliably handle large volumes of concurrent requests; performance, where increased latency arises from chaining multiple AI services and multistep reasoning; and cost, as each additional LLM call, or external tool invocation inflates operational expenses. Equally challenging are integration complexity, as agents must interoperate with heterogeneous clinical data sources and APIs; and rigorous testing and validation, which are essential to verify safety and effectiveness across diverse use cases. Adding to these complexities in agent development, recent work demonstrates that MAS do not yet deliver the substantial performance improvements often promised over single agents [56].

Furthermore, effective integration with EHR systems brings its own set of obstacles. Rheumatology data are exceptionally heterogeneous, spanning structured lab results, longitudinal composite scores, quantitative imaging, genomics, wearables and lengthy narrative notes. Robust data preprocessing pipelines, natural-language processing to structure free text and provenance-aware temporal reasoning are essential prerequisites for any rheumatology-focused agent.

## CONCLUSION

Agentic AI, by augmenting LLMs with planning, memory and the capacity for tool interaction, offers transformative potential for rheumatology, promising to overcome the limitations of current models in managing complex, data-rich clinical scenarios. This perspective has discussed the core principles of AI agents, their emergent applications across medical specialities, and significant opportunities within rheumatology, ranging from personalised treatment recommendations to automated clinical trial matching and longitudinal flare prediction. However, an AI agent is only as powerful as the set of tools and data sources it can access, the quality of which directly governs the reliability of its recommendations.

Therefore, as these systems are developed, responsible AI practices and stringent data-protection frameworks are more relevant than ever to ensure that sensitive patient information flowing between diverse sources remains secure and compliant. Despite the considerable promise, the successful deployment of agentic systems for the management of RMDs requires proactively addressing these regulatory, ethical and technical challenges. Responsible innovation and robust governance are crucial to fully realise AI’s potential to improve patient outcomes and advance the field.

## Competing interests

AMG works as a data scientist at Roche. DB has received payment honoraria for lectures, presentations, speakers bureaus or support for meeting attendance from AbbVie, Galapagos, Janssen, UCB, Pfizer and Novartis, support for attending meetings from UCB, Novartis and AbbVie. He works part-time as a senior medical advisor at Savana Research, a company on AI in medicine.

## Appendix Supplementary materials

Image, application 1

## Footnotes

Supplementary material associated with this article can be found, in the online version, at doi:10.1016/j.ero.2025.06.012.
