# Supplementary material for: From chat to act: large language model agents and agentic AI as the next frontier of AI in rheumatology
Source: EULAR Rheumatol Open. 2025 Jul 29;1(3):147–56. doi: 10.1016/j.ero.2025.06.012 (PMC13292459; doi:10.1016/j.ero.2025.06.012)
Supplement: Supplementary file 1 [file mmc1.docx]

**Supplementary Material**

**From Chat to Act: Large Language Model Agents and Agentic AI as the Next Frontier of Artificial Intelligence in Rheumatology**

Alfredo Madrid-García^1,*^, Diego Benavent^2^, Beatriz Merino-Barbancho^3, *^

^1^ Independent researcher

^2^ Rheumatology Department, Hospital Universitari de Bellvitge, Barcelona, Spain

^3^ Escuela Técnica Superior de Ingenieros de Telecomunicación. Universidad Politécnica de Madrid. Avenida Complutense, Madrid, Spain

*** Corresponding author details:**

**Alfredo Madrid García, PhD**

e-mail: [fredymad@msn.com](mailto:fredymad@msn.com)

**ORCiD**

Alfredo Madrid-García: 0000-0002-1591-0467

Diego Benavent: 0000-0001-9119-5330

Beatriz Merino-Barbancho: 0000-0001-5070-4178

##

### Architecture of AI agents

There is currently no consensus on a standard architecture for AI agents; various authors have proposed different core components and structures. Some suggest that an agent comprises multiple modules such as perception modules for interpreting multimodal data, interaction modules that engage with the environment, memory modules for storing and retrieving knowledge, and reasoning modules for planning and decision-making [1]. It has been proposed that an agent is composed of a central program, an LLM plus instructions that govern its dynamic behaviour, along with trainable decision-making agent modules such as memory, reasoning, planning, and profiling [2]. These agent modules are used by the agent to decide which action to execute by calling a tool from the environment. Perception, in this view, is part of the environment as it is how the agent interacts with it and is not a trainable decision. Other authors identified five core items in agentic AI: reasoning, memory, tools, interaction and evaluation [3]. In healthcare, a four-module architecture including profile, clinical planning, medical reasoning, and external capacity enhancement has been proposed [4]. For clarity and brevity, the present review adopts a previously described formulation that organises an AI agent into three principal components [5], see **Figure 1.**

#### Model

The decision‐making core, router, is instantiated by one or more LLMs of arbitrary size (i.e., any number of parameters) [6]. At each decision point, the router selects the next execution path. During an agent’s run, it may repeatedly invoke this router, each time supplying newly acquired context. The router then integrates that context with its internal representation of available next steps and chooses the subsequent action. Although the router is typically not pre-trained on the specific data formats or signatures of the tools it orchestrates, its accuracy can be substantially improved by providing illustrative examples. In this context, prompt‐engineering helps steer the router toward more accurate tool selection and error-free execution [7].

#### Tools

Tools are the external interfaces that let LLM reach beyond its internal knowledge and interact with the real world. These tools can be simple connectors, such as APIs, which allow LLMs to query various external services. For instance, LLMs can utilize these tools to retrieve clinical trials information (e.g., *ClinicalTrials.gov*), research papers (e.g., *PubMed Central*), current clinical decision support (e.g., *UpToDate*), consumer health information (e.g., *MedLinePlus*), drug safety or regulatory data (e.g., *OpenFDA*), and specialised evidence-retrieval platforms (e.g., OpenEvidence, Elicit), as well as web search capabilities for accessing up-to-date information on the internet. Other tools include machine learning models, code interpreters for executing algorithms; database connectors for reading EHR data; local utilities for performing domain-specific computations such as scoring of composite measures; integration frameworks that orchestrate complex workflows across multiple systems and data sources; and hardware-interface modules for interacting with physical devices [8].

Because each tool effectively enlarges the agent’s action space (i.e., complete set of operations the agent is permitted to take at any decision step), the agent must learn to propose, evaluate, and select the optimal tool chain for a given task often combining several tools sequentially such as retrieving patient history, querying a drug interaction API, and then generating a treatment plan. Furthermore, different strategies exist to teach the agent how to choose the right tools such as in-context learning, retrieval-based in-context learning, fine-tuning based learning. When orchestrated well, tool-augmented agents enable real-time decision support, and interaction with connected medical devices, overcoming the limitations of standalone LLMs.

#### Orchestration layer

The orchestration layer manages the overall workflow and interaction between the model and the tools. This layer contains two main components:

- Memory module: Memory enables an agent to sustain coherent interactions and make informed decisions and actions based on past experiences and the current context. As the agent encounters new situations and gathers data, its memory module can be updated with the latest information. Two memory types stand out. First, short-term memory, which temporarily stores information during interactions (e.g., in a conversational chatbot) and retains recent inputs to support immediate decision-making. Second, long-term memory serves as the agent’s persistent knowledge base, retaining information across sessions to support accumulated expertise. This information can be stored in an external database.
- Reasoning and planning: Reasoning is the process of making decisions based on the available information. Reasoning has been classified into two categories depending on whether agents adjust their plan in response to experimental or human feedback, direct reasoning and reasoning with feedback [1], or in three types of reasoning, namely inductive, deductive and abductive [8]. Some of the most widely used reasoning strategies include Chain-of-Thought, ReAct and Reflexion.
  - Chain-of-Thought (CoT) [9]: CoT guides a model to tackle complex problems by deconstructing them into a sequence of smaller and more manageable steps. This enables the model to develop its reasoning incrementally instead of trying to find the solution in a single leap, as it can be in the example illustrated in **Supplementary Table 1**. By following this intermediate reasoning steps CoT reduces hallucinations, improves the model's performance and enhances interpretability.
  - ReAct [10]: ReAct combines reasoning and acting. The model first generates an explicit thought that decomposes the task, then executes the corresponding action, and immediately observes the outcome before iterating. This interleaving of reasoning, acting, and post-action observation enables the system to form and update structured plans, integrate external knowledge sources, and progressively refine its decisions. **Supplementary Table 2** illustrates an example with antibodies in vasculitis.
  - Reflexion [11]: Reflexion enhances agent performance by incorporating an explicit self-reflection and evaluation phase. After a sequence of actions, the agent assesses its trajectory and the quality of its reasoning, often utilizing its memory modules to review past steps and outcomes, identifying flaws, inefficiencies, or deviations in its preceding thought processes. Based on this evaluation, the agent can then refine its strategy, adjust its plan for subsequent steps, and essentially learn from its own experience within a task to improve its decision-making over time. For instance, an agent tasked with drafting a treatment plan for a patient with difficult-to-treat lupus might initially suggest a standard therapy. Upon reflection, it might then refine the plan to include a more novel or combination therapy, justifying the change based on this reflective analysis.

A primary function of this layer is to interpret the user's query to select the most appropriate tool for the task. It must then handle the composition of different tools. It must understand the required input format for each tool and rely on a predictable output format from them. This predictability is essential, as it allows the orchestration layer to seamlessly use the output of one tool as the input for the next, enabling complex, multi-step task execution.

## AI agents in rheumatology

**Supplementary Table 3** shows additional applications of AI agents in rheumatology

## References

1 Gao S, Fang A, Huang Y, *et al.* Empowering biomedical discovery with AI agents. *Cell*. 2024;187:6125–51. doi: 10.1016/j.cell.2024.09.022

2 Ramos MC, Collison CJ, White AD. A review of large language models and autonomous agents in chemistry. *Chem Sci*. 2025;16:2514–72. doi: 10.1039/D4SC03921A

3 Schneider J. Generative to Agentic AI: Survey, Conceptualization, and Challenges. Published Online First: 26 April 2025.

4 Wang W, Ma Z, Wang Z, *et al.* A Survey of LLM-based Agents in Medicine: How far are we from Baymax? 2025.

5 Wiesinger J, Marlow P, Vuskovic V. Agents. Google. 2024.

6 Matarazzo A, Torlone R. A Survey on Large Language Models with some Insights on their Capabilities and Limitations. Published Online First: 3 January 2025.

7 Venerito V, Lalwani D, Del Vescovo S, *et al.* Prompt engineering: The next big skill in rheumatology research. *Int J Rheum Dis*. 2024;27. doi: 10.1111/1756-185X.15157

8 Biswas A, Talukdar W. *Building Agentic AI Systems: Create intelligent, autonomous AI agents that can reason, plan, and adapt*. 1st ed. Packt Publishing 2025.

9 Wei J, Wang X, Schuurmans D, *et al.* Chain-of-Thought Prompting Elicits Reasoning in Large Language Models. 2022.

10 Yao S, Zhao J, Yu D, *et al.* ReAct: Synergizing Reasoning and Acting in Language Models. 2022.

11 Shinn N, Cassano F, Berman E, *et al.* Reflexion: Language Agents with Verbal Reinforcement Learning. 2023.

## Supplementary tables

**Supplementary Table 1:** Example of Chain-of-Thought for clinical decision in rheumatoid arthritis

| **CoT step** | **Clinical information identified** | **Evidence-based interpretation** | **Consequent recommendation** |
| --- | --- | --- | --- |
| **1** | Morning stiffness > 60 min and symmetric MCP swelling | High pre-test probability of early RA | Proceed to further evaluation |
| **2** | Positive anti-CCP antibodies | > 95 % specificity for RA; favours inflammatory arthritis over osteoarthritis | RA suspicion consolidated |
| **3** | Need for timely management | Delayed treatment worsens outcomes; EULAR advises referral ≤ 6 weeks from symptom onset | Urgent referral warranted |

CoT: Chain-of-Thought; MCP: metacarpophalangeal; RA: rheumatoid arthritis

**Supplementary Table 2**. ReAct workflow for Identifying c-ANCA (PR3-ANCA) in granulomatosis with polyangiitis

| Step | Type | Detail |
| --- | --- | --- |
| 1 | Thought | I need to identify the auto-antibody that is classically associated with GPA. First, I should look up “Granulomatosis with Polyangiitis auto-antibody”. |
| 2 | Act | Search [Granulomatosis with Polyangiitis auto-antibody] |
| 3 | Obs | Results mention that the c-ANCA, directed against PR3, is strongly linked to GPA. |
| 4 | Thought | To be sure c-ANCA/PR3-ANCA is indeed the key finding, I’ll quickly cross-check “PR3-ANCA disease association”. |
| 5 | Act | Search [PR3-ANCA disease association] |
| 6 | Obs | Sources confirm PR3-ANCA (c-ANCA pattern on immunofluorescence) is characteristic of GPA and helps distinguish it from other vasculitides. |
| 7 | Thought | Both searches converge on the same antibody. That’s sufficient. |
| 8 | Act | Finish [c-ANCA (PR3-ANCA)] |

GPA: Granulomatosis with Polyangiitis; cytoplasmic ANCA (c-ANCA); proteinase-3 (PR3)

**Supplementary Table 3:** Other potential applications of AI agents in rheumatology

| **Application** | **Description** |
| --- | --- |
| Diagnostic support | An AI agent could be equipped with multimodal perception tools capable of synthesizing patient history, physical-exam findings, serologies, and imaging to generate differential diagnoses.   - **Symptom clustering & pattern recognition**: By extracting features (e.g., morning stiffness duration, joint distribution, serologic markers (RF, anti-CCP), inflammatory indices (ESR/CRP)) the agent might rank the likelihood of RA, PsA, crystal arthropathies or mimics. - Employing a ReAct framework, the agent could query external knowledge bases (e.g., UpToDate, PubMed) to validate uncommon presentations (e.g., seronegative RA with erosions) before returning a confidence-scored diagnosis list. |
| Personalized treatment recommendation | Leveraging guideline‐encoded decision logic (ACR/EULAR), EHR data, pharmacogenomic profiles, and up-to-date trial results from ClinicalTrials.gov and PubMed, an AI agent could propose individualized treatment pathways under Treat-to-Target principles.   - Comorbidity and safety filtering: The agent’s toolchain might include a drug–drug and drug–disease interaction API (e.g., OpenFDA, DrugBank, RxNorm) to exclude contraindicated DMARDs in patients with liver disease or heart failure - Adaptive planning: Through a reflexion module, the agent might reassess treatment efficacy at each follow-up by analysing trends in DAS28, SLEDAI or BASDAI, potentially recommending dynamic dose adjustments or therapy switches. |
| Patient Monitoring & Management | Through integration of wearable-device feeds and PROMs, an AI agent might continuously assess disease activity and adherence.   - Flare prediction: A trained time-series model within the agent’s toolset could flag patterns (e.g., decreasing step count, worsening pain scores) that precede psoriatic arthritis flares, triggering automated clinician alerts. - Engagement nudges: When adherence thresholds fall below agreed targets, the agent could dispatch personalized reminders or educational content via patient portals, documenting each interaction in the EHR. |
| Clinical trials | An eligibility-screening agent could parse EHR demographics, lab results, imaging reports and free-text notes to match patients to active trials.   - Complex criteria handling: Using CoT reasoning, the agent could walk through inclusion/exclusion rules (e.g., minimum disease duration, prior biologic exposures, organ-function thresholds) then output a shortlist of trials with links and contact information. - Automated outreach: Upon patient consent, the agent could generate pre-populated referral forms or notifications to study coordinators, vastly reducing screening workload. |
| Administrative task automation & care coordination | - Multi-Agent care teams: Specialized agents representing the rheumatologist, dermatologist, physical therapist, and primary care physician could coordinate through a shared memory layer. They might negotiate appointment sequencing, reconcile medication plans, and ensure that psoriatic arthritis patients receive skin assessments, joint injections, and exercise prescriptions in a unified care pathway. |

ACR: American College of Rheumatology; AI: Artificial intelligence; anti-CCP: Anti-cyclic citrullinated peptide antibody; API: Application programming interface; BASDAI: Bath Ankylosing Spondylitis Disease Activity Index; CoT: Chain-of-thought reasoning; CRP: C-reactive protein; DAS28: 28-Joint Disease Activity Score; DMARDs: Disease-modifying antirheumatic drugs; EHR: Electronic health record; ESR: Erythrocyte sedimentation rate; EULAR: European Alliance of Associations for Rheumatology; FDA: Food and Drug Administration; PROMs: Patient-reported outcome measures; PsA: Psoriatic arthritis; RA: Rheumatoid arthritis; ReAct: Reason + Act framework; RF: Rheumatoid factor; SLEDAI: Systemic Lupus Erythematosus Disease Activity Index.
